# Ginsenoside Rh3 Inhibits Lung Cancer Metastasis by Targeting Extracellular Signal-Regulated Kinase: A Network Pharmacology Study

**DOI:** 10.3390/ph15060758

**Published:** 2022-06-17

**Authors:** Xiaodan Xue, Yannan Liu, Linlin Qu, Cuiying Fan, Xiaoxuan Ma, Pingkai Ouyang, Daidi Fan

**Affiliations:** 1 Shaanxi Key Laboratory of Degradable Biomedical Materials, School of Chemical Engineering, Northwest University, Taibai North Road 229, Xi’an 710069, China; xuexiaodan@stumail.nwu.edu.cn (X.X.); liuyannan@nwu.edu.cn (Y.L.); linlinqu1994@163.com (L.Q.); xiaoxuanma@nwu.edu.cn (X.M.); 2 Shaanxi R & D Center of Biomaterials and Fermentation Engineering, School of Chemical Engineering, Northwest University, Taibai North Road 229, Xi’an 710069, China; 3 Biotech. & Biomed. Research Institute, Northwest University, Taibai North Road 229, Xi’an 710069, China; 4 Xi’an Giant Biotechnology Co., Ltd., Xi’an 710076, China; wangyoucao526@163.com; 5 College of Biotechnology and Pharmaceutical Engineering, Nanjing Tech University, Nanjing 211816, China; ouyangpk@njtech.edu.cn

**Keywords:** ginsenoside Rh3, network pharmacology, human lung cancer, metastasis, ERK

## Abstract

Lung cancer has a high mortality rate and is very common. One of the main reasons for the poor prognosis of patients with lung cancer is the high incidence of metastasis. Ginsenoside Rh3, a rare ginsenoside extracted from Panax notoginseng, exhibits excellent anti-inflammatory and anti-tumor effects. Nonetheless, the inhibitory potential of Rh3 against lung cancer remains unknown. The target genes of Rh3 were screened by the PharmMapper database; the proliferation of lung cancer cells was detected by MTT assay; the migration and invasion of cells were detected by the Transwell method; and the expression of extracellular signal-regulated kinase (ERK) and EMT-related proteins in vivo and in vitro were detected by Western blotting. In addition, we established a lung metastasis model in nude mice using A549 cells to assess the effect of Rh3 on NSCLC tumor metastasis in vivo. Our findings suggest that Rh3 significantly inhibited lung cancer metastasis both in vivo and in vitro. It was determined by flow cytometry analysis that Rh3 notably inhibited cell proliferation by blocking the G1 phase. In addition, Rh3 inhibited metastasis in lung cancer cells and regulated the expression of metastasis-related proteins under hypoxia. Mechanistic studies suggested that Rh3 targeted ERK to inhibit lung cancer metastasis. The ERK inhibitor U0126 or siRNA-mediated knockdown of ERK had an enhanced effect on Rh3’s ability to inhibit lung cancer metastasis. The studies revealed that the inhibitory effect of Rh3 on the metastatic ability of lung cancer cells may be supported by ERK-related signaling pathways.

## 1. Introduction

Lung cancer has a high mortality rate and is very common. It is second only to breast cancer in terms of estimated morbidity; however, the mortality rate ranks first among all cancers [1]. Small cell lung cancer (SCLC) and non-small cell lung cancer (NSCLC) are two forms of lung cancer, accounting for 15% and 85% cases, respectively. One of the main reasons for the poor prognosis of patients with lung cancer is the high incidence of metastasis [2,3]. The exact mechanism of tumor metastasis remains unclear because of the intricacy of the process. Epithelial–mesenchymal transition (EMT) plays an essential role in promoting tumor migration and invasion [4,5,6,7]. Although the mortality rate of lung cancer has decreased, it is still a fatal illness. Therefore, to enhance the survivability of lung cancer patients, there is a pressing need to expand novel treatment strategies.

Hypoxia is associated with the metastasis of tumors and is a common phenomenon in human tumors [8,9]. During the process of tumorigenesis, hypoxia promotes the multiplication of tumor cells and angiogenesis. Hypoxia affects multiple signaling pathways in cells, including hypoxia-inducible factor (HIF) and extracellular signal-regulated kinase (ERK) pathways, which regulate apoptosis, proliferation, metastasis, and inflammation in cancer cells [10,11]. The ERK signaling pathway is closely related to tumor migration and invasion [12,13]. Phosphorylated ERK will subsequently activate multiple processes involved in cell proliferation, motility, and metastasis [14]. For instance, direct phosphorylation of ERK causes HIF-1α to remain in the nucleus, allowing for its accumulation and activation in the nucleus [15]. One of the important target genes of HIF-1α is vascular endothelial growth factor (VEGF), which is associated with EMT. The binding of VEGF to its receptor results in the autophosphorylation of specific tyrosine residues in the structural region of the cytoplasm, which is inextricably linked to cell proliferation and migration [16,17]. Gefitinib has been shown to inhibit the ability of non-small cell lung cancer to metastasize by modulating the EMT process [18]. Gefitinib also has a regulatory effect on the ERK signaling pathway, thereby inhibiting cancer cell growth and metastasis [19,20]. Consequently, inhibition of tumor cell metastasis through interaction with ERK is regarded as an effective strategy to prevent and treat lung cancer.

Network pharmacology is supported by bioinformatics and systems biology [21]. It helps discover potential mechanisms involving drug compounds and their putative targets by analyzing the compositions of drugs and their targets and pathways [22]. Interestingly, certain active ingredients of natural origin have been reported to possess pharmacological activity with high efficacy in treating several diseases [23]. Based on their selective molecular targets, these bioactive components can act as novel, reliable, and effective therapeutic elements in treating different types of human cancers [24,25].

Ginseng is a well-known traditional herbal medicine. Ginseng, which includes Asian ginseng (Panax ginseng C. A. Meyer) and American ginseng (*P. quinquefolius* L.), is grown in large quantities and is highly valued as a traditional medicinal plant in China, Russia, Korea, and other countries. Ginseng has been used since ancient times to treat various diseases and stress induction, improve diabetes, enhance physical performance, and for its fatigue-reducing and anti-aging effects [26,27]. Despite its long growth cycle, it has a wide range of pharmacological activities and is therefore of great interest. The pharmacological properties of ginseng are largely attributed to its active ingredients ginsenosides [28]. Ginsenosides have a variety of pharmacological activities, such as anti-tumor [29], anti-inflammatory [30], anti-diabetic [31], anti-apoptotic [32], and neuroprotective effects [33], as well as therapeutic properties against cardiovascular diseases. Ginsenoside Rh3 consists of a tetracyclic triterpenoid aglycon and a glycoside, which is a triterpenoid saponin. Rh3 is a bacterial metabolite of ginsenoside Rg5 [34]. Recent studies have demonstrated that Rh3 possesses several useful properties, such as anti-inflammatory [35], antioxidant damage [36], anti-apoptotic [37], and anti-tumor effects. However, whether Rh3 has an inhibitory effect on NSCLC metastasis is a question that remains unexplored. In this research, we examined the potential of Rh3 to inhibit metastasis of lung cancer cells both in vitro and in vivo.

## 2. Results

### 2.1. Inhibition of Lung Cancer Growth by Ginsenoside Rh3 In Vitro

**Figure 1:**
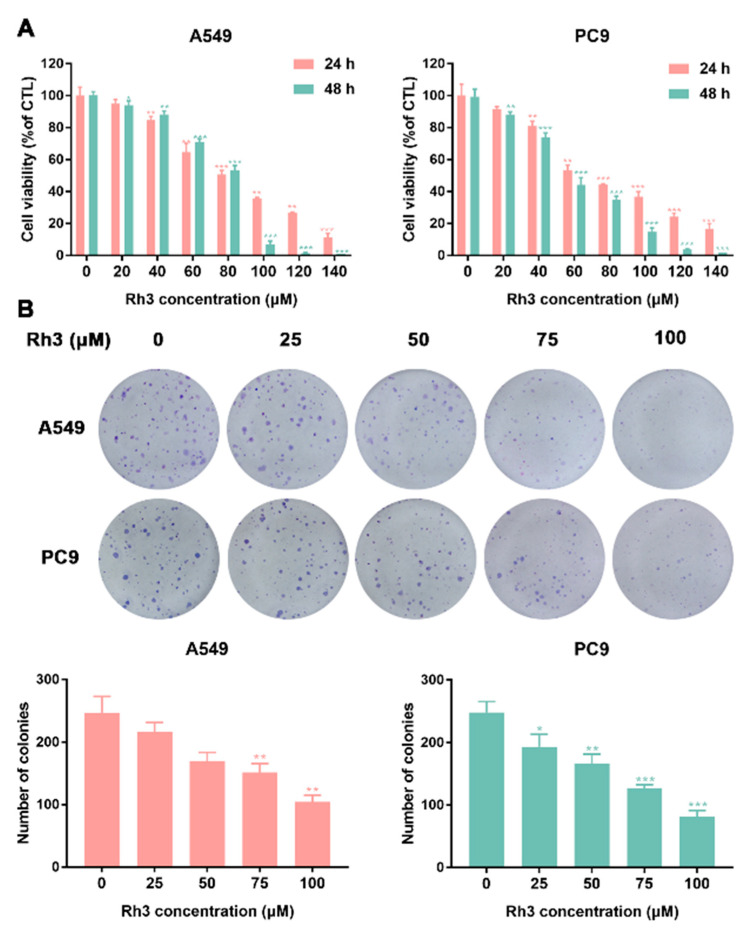
Ginsenoside Rh3 suppresses the growth of lung cancer cells in vitro. (**A**) Human lung cancer cells (A549 and PC9) were treated with different concentrations (0–140 µM) of Rh3 for 24 h or 48 h. Cell viability was measured by MTT assay. Rh3 suppresses the proliferation of A549 and PC9 cells in a dose- and time-dependent manner. (**B**) Colony formation assay in A549 and PC9 cells with control or Rh3 (25–100 µM) treatment. * *p* < 0.05, ** *p* < 0.01, *** *p* < 0.01 compared with the control.

To investigate the inhibitory effect of Rh3 on the proliferation ability of lung cancer cells, a cytotoxic assay (MTT assay) was performed by incubating A549 and PC9 cells with various concentrations of Rh3 for 24 or 48 h. The results showed that Rh3 inhibited the proliferation of A549 and PC9 cells in a time- and dose-dependent manner (Figure 1A).

Next, a colony formation assay was used to examine the impact of Rh3 on the proliferation capacity of A549 and PC9 cells. As Figure 1B shows, in comparison with the control group, treatment with different concentrations of Rh3 (0–25 μM) did not exert significant effects on colony formation. The concentration of Rh3 above 50 μM exerted a significant effect on the proliferation capacity of A549 and PC9 cells. The above-mentioned data suggested that Rh3 suppressed the proliferation of human lung cancer cells in a time- and concentration-dependent manner.

### 2.2. Ginsenoside Rh3 Triggers G1 Phase Arrest In Vitro

**Figure 2:**
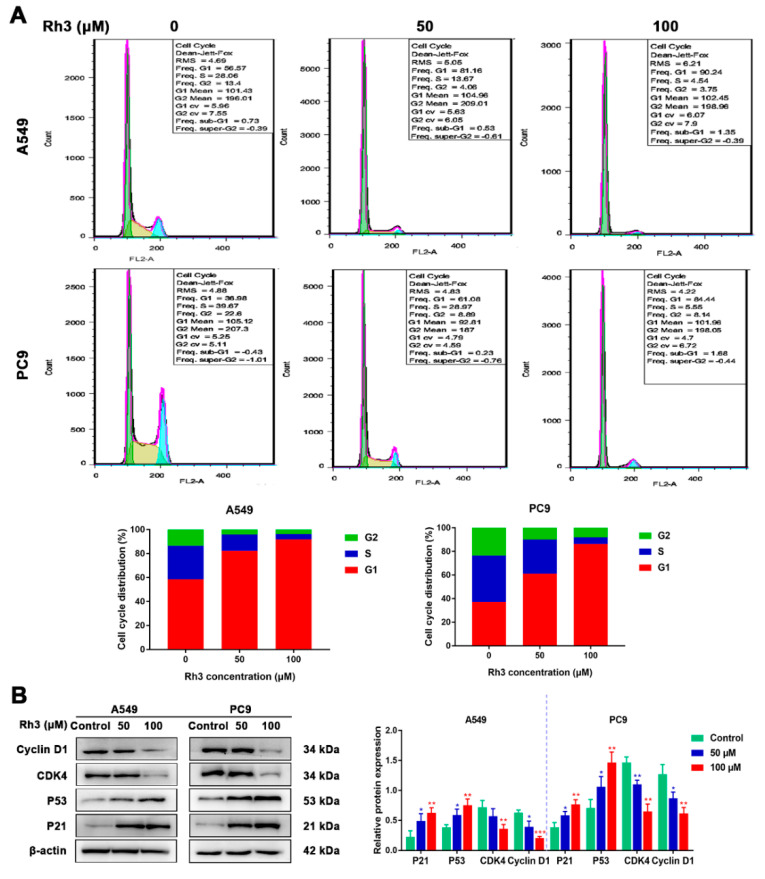
Ginsenoside Rh3 induces G1 cell cycle arrest in human lung cancer cells. (**A**) Rh3 (50 and 100 µM) induced G1 cell cycle arrest. Lung cancer cells were treated with Rh3 for 24 h and analyzed by flow cytometry. (**B**) Western blotting shows the levels of G1-related proteins of lung cancers after Rh3 (50 and 100 µM) treatment. β-actin was used as an endogenous reference. The histogram represents the statistical analysis of the relative expression of G1-related proteins. * *p* < 0.05, ** *p* < 0.01, *** *p* < 0.001 compared with the control.

The impact of Rh3 on the cell cycle of A549 and PC9 cells was investigated by flow cytometry. As Figure 2A shows, contrasted with the control group, the proportion of G1-phase cells markedly increased and those of S-phase and G2-phase cells decreased after Rh3 treatment. In addition, we used Western blotting to measure the presence of cell cycle-related protein expression. The experimental findings showed that Rh3 significantly reduced the expression of CyclinD1 and cyclin-dependent kinase 4 (CDK4) and increased the expression of p21 and p53 dose-dependently (Figure 2B). Altogether, Rh3 inhibits the growth of tumors in vitro through blockade of G1-phase cells.

### 2.3. Ginsenoside Rh3 Inhibits Hypoxia-Induced Migration and Invasion in Lung Cancer Cells

**Figure 3:**
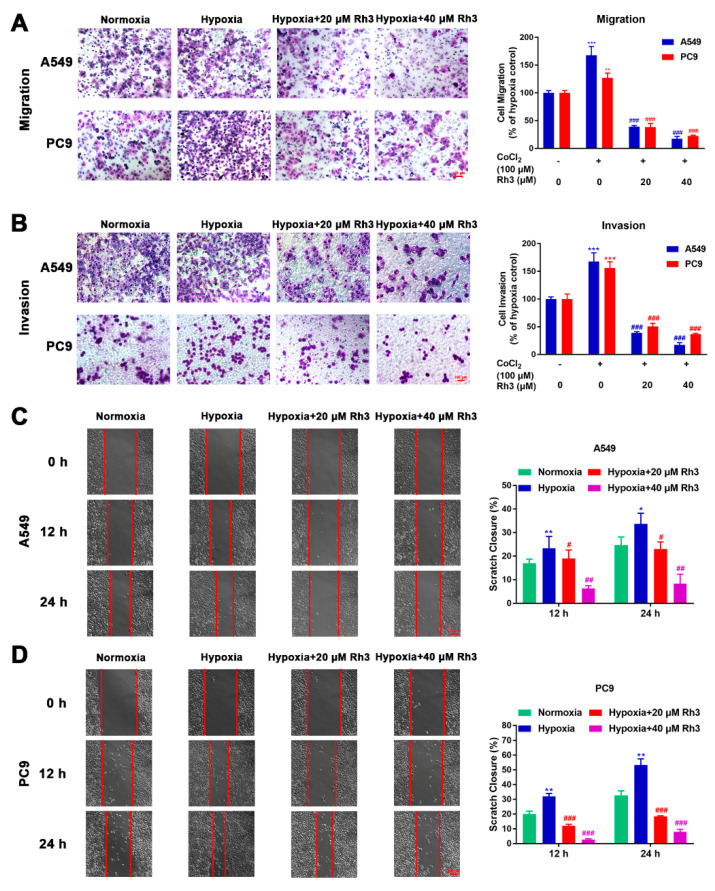
Rh3 inhibits the metastasis of lung cancer cells. The impact of Rh3 (20 and 40 µM) on metastasis potential in A549 and PC9 cells as determined by Transwell assay (**A**,**B**) and wound-healing assay (**C**,**D**). Cells had been incubated under normoxia or hypoxia, induced by CoCl_2_, and treated with different doses of Rh3. Crystal violet was used to stain migrated and invaded cells in the Transwell assay; the data are expressed as percentages of CoCl_2_-treated cells. For the cell wound-healing assay, at 12 h and 24 h after scratching, the area of the wound area was assessed by microscopy at 100× magnification. * *p* < 0.05, ** *p* < 0.01, *** *p* < 0.001 compared with the control. # *p* < 0.05, ## *p* < 0.01, ### *p* < 0.001 compared with control cells treated with CoCl_2_.

We explored the migration and invasion capacity of lung cancer cells through wound healing and Transwell assays. CoCl_2_ was used to simulate in vitro hypoxic conditions. The cytotoxicity of CoCl_2_ is shown in Figure S1, and we finally chose a concentration of 100 μM to induce hypoxia. For the migration assay, as shown in Figure 3A, the migration rates for A549 and PC9 cells treated with CoCl_2_ were enhanced by 36% and 25%, respectively, compared with the control cells. Compared with CoCl_2_ treatment alone, Rh3 inhibited metastatic capacities at 63–85% and 68–79% in A549 and PC9 cells (after induced hypoxia).

For the invasion experiment, the percentage of invasion of A549 and PC9 cells was observed to be 36.4% and 40.54% higher, respectively, under hypoxic conditions compared to normoxic conditions. We further investigated whether Rh3 depressed the invasive capability of hypoxia-induced cells. As compared with the induced hypoxia group alone, the invasive capability of hypoxia-induced cells was suppressed by Rh3, decreasing by 64.1–79.2% and 52.03–70.63% among A549 and PC9 cells, respectively (Figure 3B). The outcomes showed that Rh3 hindered invasion ability.

In the wound-healing assay, cells were coped with CoCl_2_ (100 μM), the least toxic concentration for lung cancer cell lines, followed by treatment with Rh3 (20 μM and 40 μM). The results showed that hypoxic conditions of cell culture markedly enhanced cell migration compared with normoxic cell culture conditions. In A549 and PC9 cells, this induction was limited to a concentration-dependent mode upon Rh3 processing (Figure 3C,D).

### 2.4. Regulation of Lung Cancer Metastasis-Associated Proteins by Rh3

**Figure 4:**
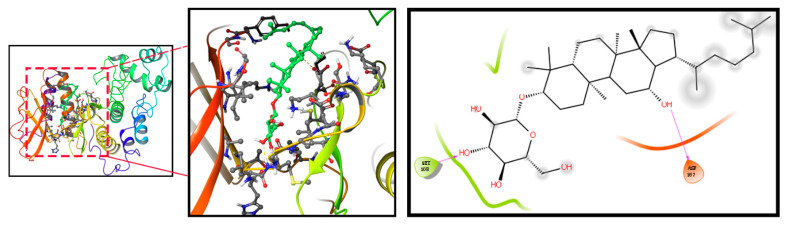
Molecular docking simulation results for Rh3 with ERK.

**Figure 5:**
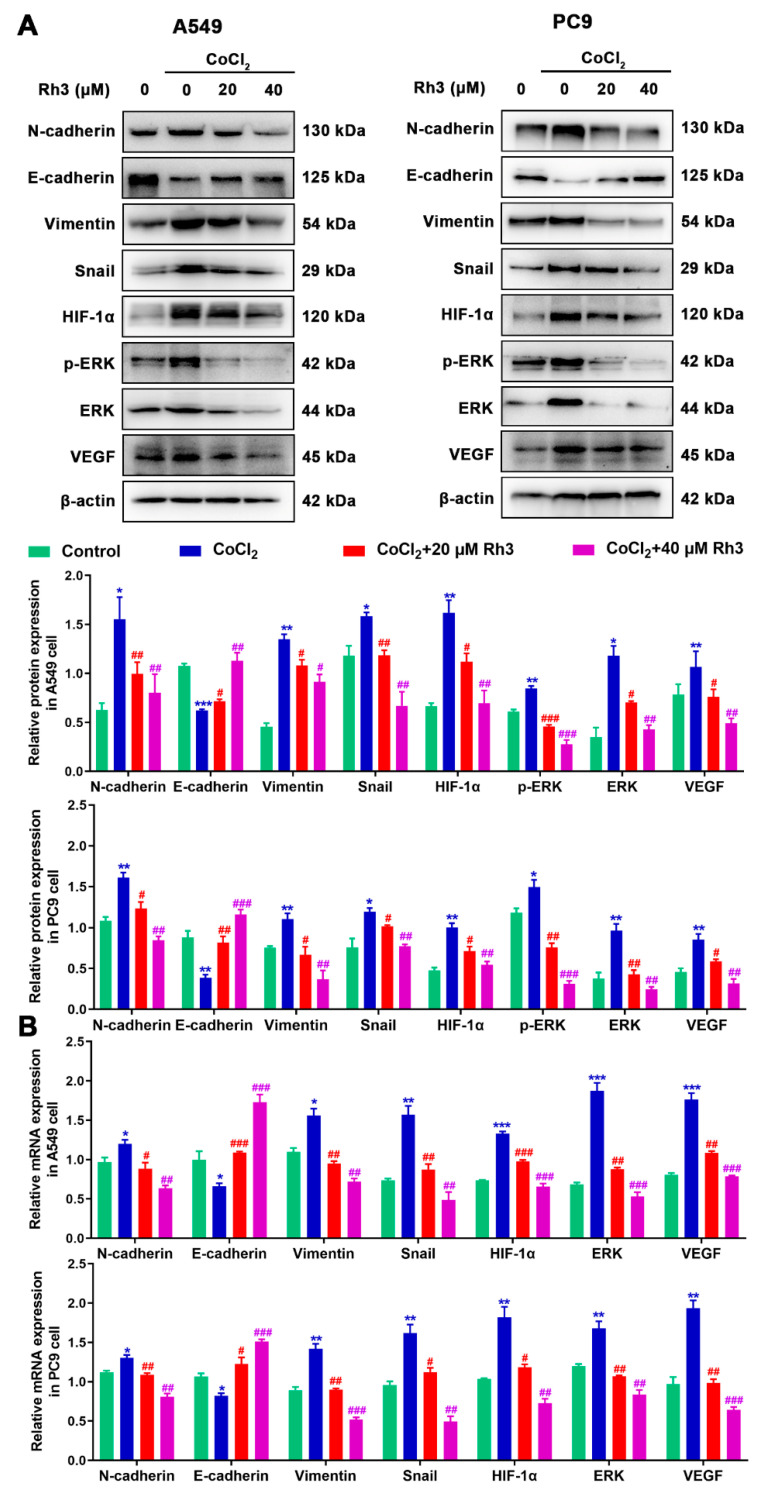
Rh3 (20 and 40 µM) inhibits EMT progression via the ERK pathway in A549 and PC9 cells. Treatment of the cells with 100 μM CoCl_2_ for 24 h. Western blotting (**A**) and qRT-PCR (**B**) shows the expression levels of N-cadherin, E-cadherin, Vimentin, Snail, HIF-1α, p-ERK, ERK, and VEGF in A549 and PC9 cells. * *p* < 0.05, ** *p* < 0.01, *** *p* < 0.001 compared with the control; # *p* < 0.05, ## *p* < 0.01, ### *p* < 0.001 compared with control cells treated with CoCl_2_.

We firstly used PharmMapper to obtain the target gene for Rh3. Rh3 has the molecular formula C_37_H_62_O_7_, and the Rh3 structure formula is shown in Figure S2A. Protein–protein interaction (PPI) targets with high confidence scores (>0.96) were screened for the network construction (Figure S2B). The drug–potential target network constructed by Cytoscape is shown in Figure S2C. Considering that Rh3 directly targeted ERK or the complete signaling pathway, we next investigated the possible binding mechanism of Rh3 to ERK by molecular docking (Figure 4). To further investigate how Rh3 inhibited metastasis in lung cancer, we explored the transfer of related proteins and gene expression. As Figure 5A show, Rh3 regulated the expression of metastasis-associated proteins (E-cadherin, N-cadherin, Vimentin, and Snail) in lung cancer cells. In addition, the impact of Rh3 at the transcriptional levels of metastasis-associated genes was identical to that at the level of proteins. As Figure 5B show, qRT-PCR analysis showed that CoCl_2_ induction upregulated transcript expression of N-cadherin, Vimentin, and Snail, whereas it downregulated transcript levels of E-cadherin in lung cancer cells. Rh3 treatment (20 and 40 μM) downregulated levels of transcripts of N-cadherin, Vimentin, and Snail and upregulated levels of transcripts of E-cadherin in lung cancer cells. The findings confirmed the suppressive actions of Rh3 on the capability of lung cancer cells to metastasize. Interestingly, the expression of phosphorylated ERK (p-ERK) was significantly reduced after Rh3 treatment, implying that ERK is a key target for the regulation of metastasis. These results suggested p-ERK as a critical factor for Rh3 to inhibit metastasis in lung cancer cells. Altogether, Rh3 has a regulatory effect on metastasis-associated proteins in lung cancer cells. Next, the mechanism of Rh3-induced ERK activation in lung cancer was elucidated. Meanwhile, we analyzed HIF-1α, VEGF, and ERK levels and their phosphorylated forms through Western blotting. HIF-1α, VEGF, ERK, and p-ERK levels were significantly increased after CoCl_2_-induced hypoxia. Expression of HIF-1α, ERK, p-ERK, and VEGF decreased after Rh3 treatment, in contrast to the hypoxia-induced condition.

### 2.5. Ginsenoside Rh3 Targeting ERK Inhibits Metastasis Capability in Lung Cancer Cells

**Figure 6:**
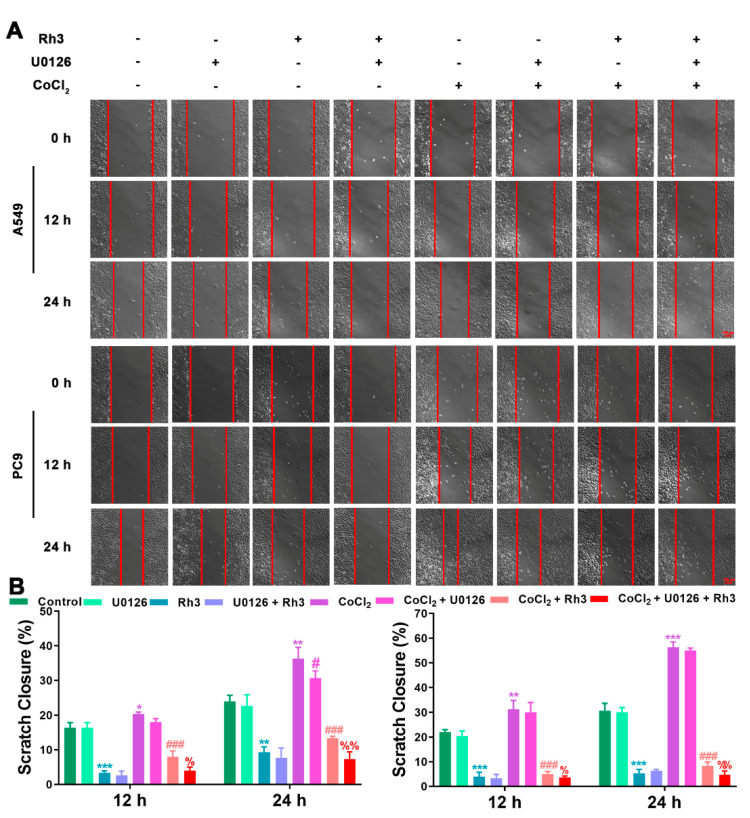
U0126 augments the inhibitory action of Rh3 in A549 and PC9 cells with respect to metastatic ability. (**A**) A549 and PC9 cells treated with 40 µM Rh3, 10 µM U0126, and 100 µM CoCl_2_ for the wound-healing assay. (**B**) The wound closures for A549 and PC9 cells at 12 h and 24 h are represented using the histogram. * *p* < 0.05, ** *p* < 0.01, *** *p* < 0.001 compared with the control group; # *p* < 0.05, ### *p* < 0.001 compared with the CoCl_2_ group; % *p* < 0.05, %% *p* < 0.01 compared with the CoCl_2_ + Rh3 group.

**Figure 7:**
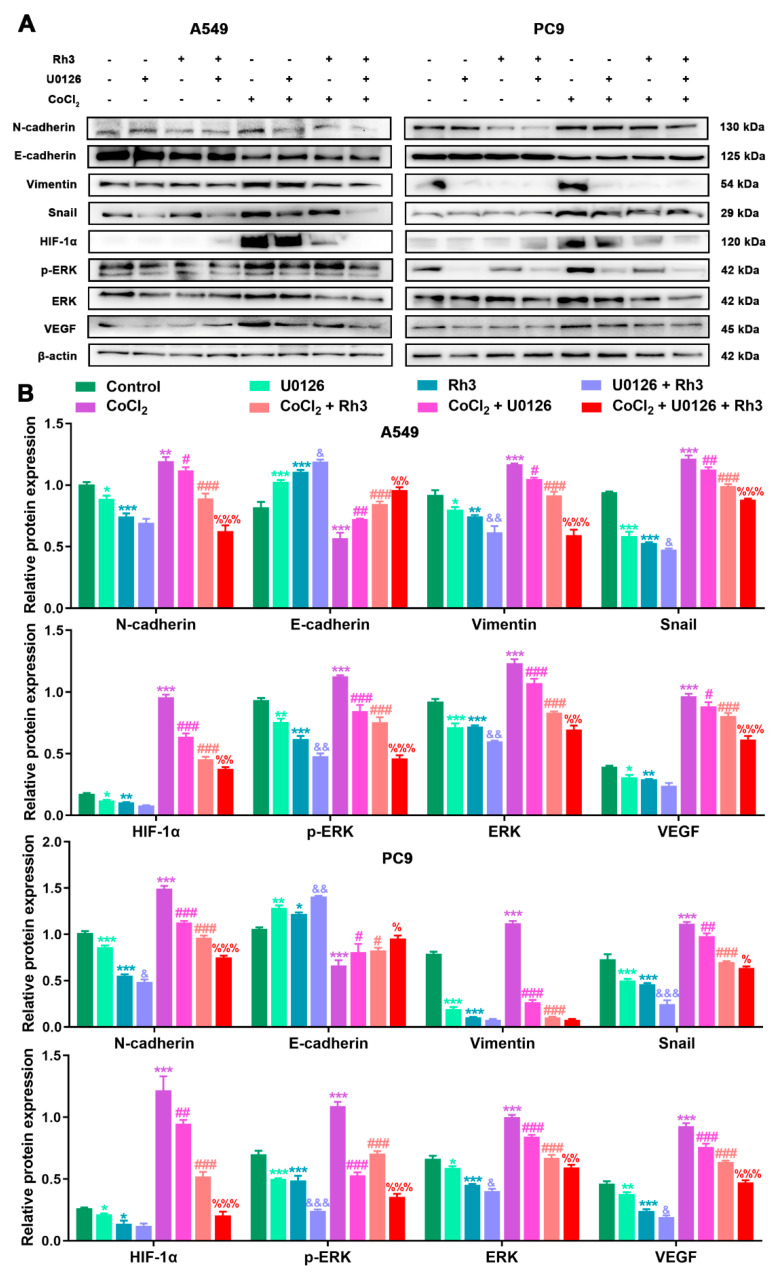
U0126 enhances Rh3 action in relation to the EMT process and the ERK pathway in A549 and PC9 cells. (**A**) HIF-1α, ERK, p-ERK, VEGF, and EMT-related protein expression in A549 and PC9 cells following treatment with 40 µM Rh3, 10 µM U0126, and 100 µM CoCl_2_. (**B**) Protein expression is represented by the histograms. * *p* < 0.05, ** *p* < 0.01, *** *p* < 0.001 compared with the control group; # *p* < 0.05, ## *p* < 0.01, ### *p* < 0.001 compared with the CoCl_2_ group; & *p* < 0.05, && *p* < 0.01, &&& *p* < 0.001 compared with the Rh3 group; % *p* < 0.05, %% *p* < 0.01, %%% *p* < 0.001 compared with the CoCl_2_ + Rh3 group.

**Figure 8:**
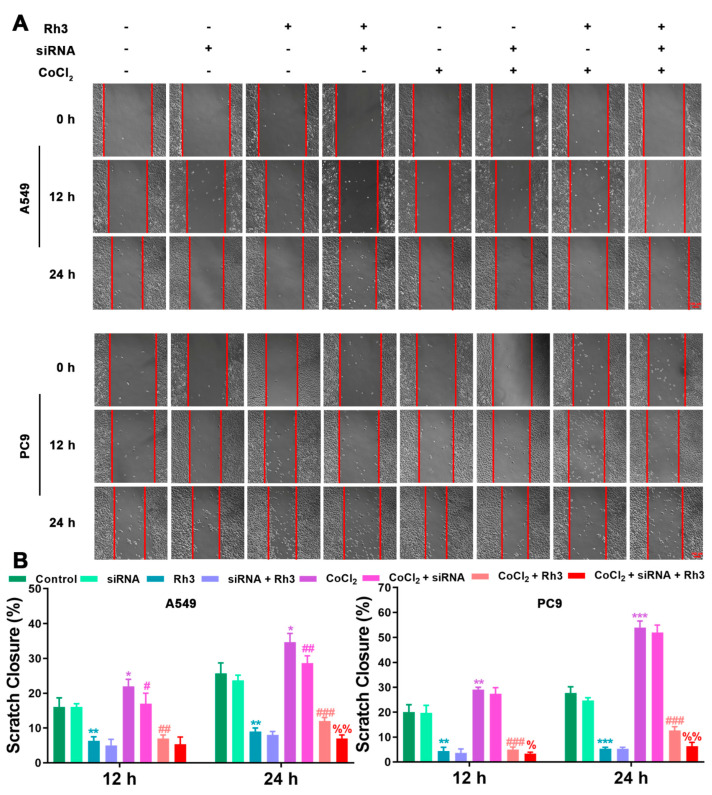
ERK-siRNA augments the inhibitory action of Rh3 in A549 and PC9 cells with respect to metastatic ability. (**A**) A549 and PC9 cells treated with 40 µM Rh3, ERK-siRNA, and 100 µM CoCl_2_ for the wound-healing assay. (**B**) The wound closures for A549 and PC9 cells at 12 h and 24 h are represented using the histogram. * *p* < 0.05, ** *p* < 0.01, *** *p* < 0.001 compared with the control group; # *p* < 0.05, ## *p* < 0.01, ### *p* < 0.001 compared with the CoCl_2_ group; % *p* < 0.05, %% *p* < 0.01 compared with the CoCl_2_ + Rh3 group.

**Figure 9:**
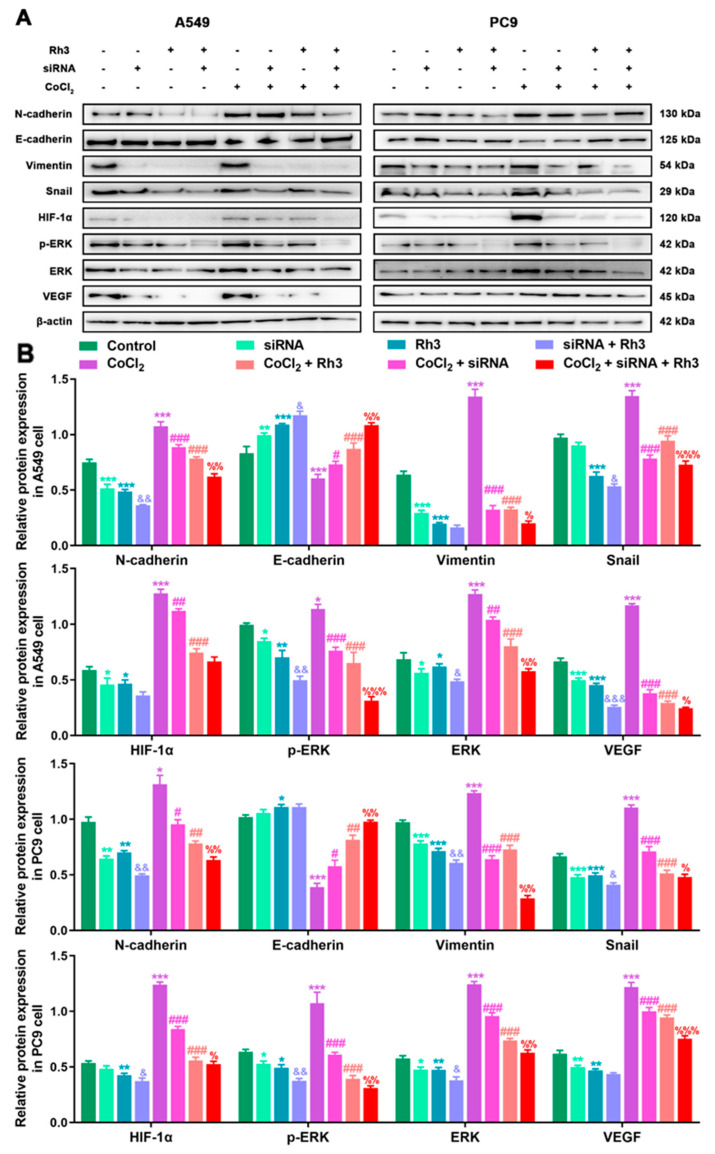
ERK-siRNA enhances Rh3 action in relation to the EMT process and the ERK pathway in A549 and PC9 cells. (**A**) HIF-1α, ERK, p-ERK, VEGF, and EMT-related protein expression in A549 and PC9 cells following treatment with 40 µM Rh3, siRNA, and 100 µM CoCl_2_. (**B**) Protein expression is represented by the histograms. * *p* < 0.05, ** *p* < 0.01, *** *p* < 0.001 compared with the control group; # *p* < 0.05, ## *p* < 0.01, ### *p* < 0.001 compared with the CoCl_2_ group; & *p* < 0.05, && *p* < 0.01, &&& *p* < 0.001 compared with the Rh3 group; % *p* < 0.05, %% *p* < 0.01, %%% *p* < 0.001 compared with the CoCl_2_ + Rh3 group.

In order to determine the mechanism of Rh3 action, in addition to whether ERK is the key target for the inhibition of tumor cell metastasis, we initially evaluated the capability of lung cancer cells to metastasize after combined treatment with Rh3 and ERK inhibitors. The analysis suggested that the metastatic abilities of A549 and PC9 cells were inhibited by combined induction with the inhibitor U0126 and Rh3, as compared with Rh3 alone (Figure 6). Furthermore, Western blotting (Figure 7) showed that U0126 reinforced the downregulation of N-cadherin, Vimentin, Snail, HIF-1α, p-ERK, ERK, and VEGF in A549 and PC9 cells after Rh3 treatment, whereas the opposite effect was observed for E-cadherin. Furthermore, we pretreated A549 cells with an siRNA against ERK to explore the mechanism by which Rh3 inhibits metastasis in lung cancer cells. As shown in Figure 8, ERK-siRNA boosted the inhibitory action of Rh3 with respect to the capacity of A549 and PC9 cells to metastasize. SiRNA-mediated downregulation of ERK significantly suppressed levels of N-cadherin, Vimentin, Snail, HIF-1α, p-ERK, ERK, and VEGF, whereas it upregulated the expression of E-cadherin (Figure 9). Thus, these results suggested a complex interaction between the EMT and ERK pathways which allows Rh3 to inhibit metastasis in lung cancer cells.

### 2.6. Rh3 Inhibits Metastasis of Lung Cancer and Triggers G1 Phase Arrest In Vivo

**Figure 10:**
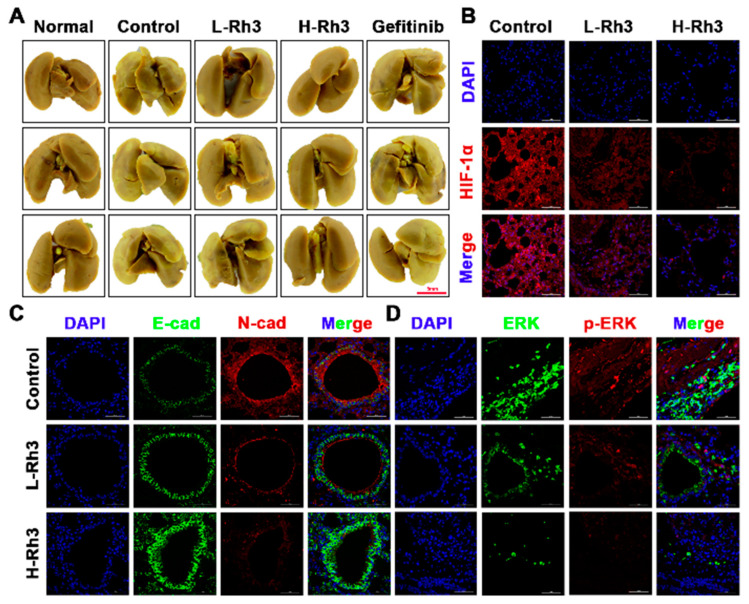
Rh3 significantly inhibited lung metastasis in a pulmonary metastasis mouse model. (**A**) Incidence of lung metastases from the same group of animals. The extent of lung metastases was quantified by staining with Bouin’s solution. (**B**) Immunofluorescence staining of HIF-1α protein in lung tissues. (**C**) Immunofluorescence staining of E-cadherin and N-cadherin proteins in lung tissues. (**D**) Immunofluorescence staining of ERK and p-ERK proteins in lung tissues. L-Rh3, 50 mg/kg; H-Rh3, 100 mg/kg; Gefitinib, 40 mg/kg.

**Figure 11:**
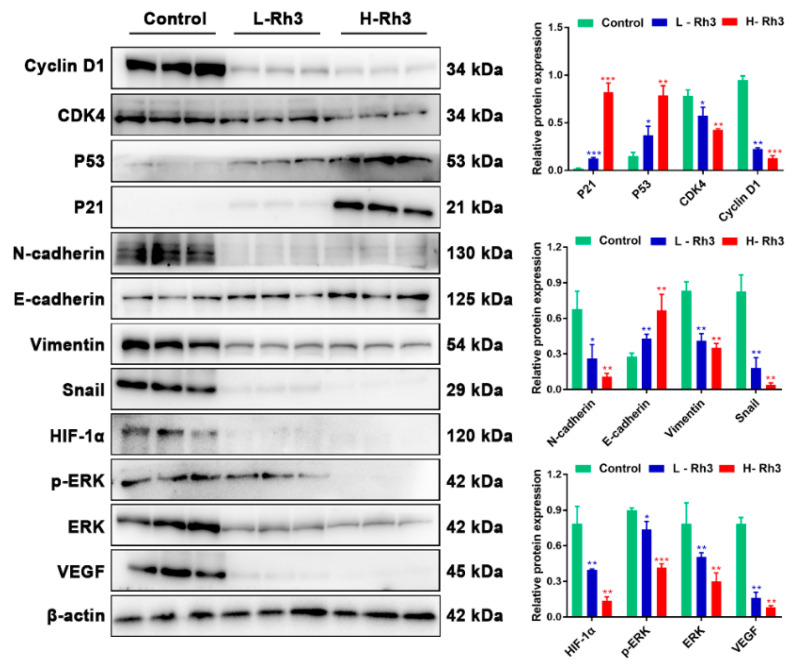
Rh3 blocked the growth of lung cancer in the G1 phase and inhibited the EMT process in the pulmonary metastasis mouse model. Western blotting detection of G1 phase-, EMT-, and ERK-related proteins in lung tissues. Data are presented as means ± standard deviations (SDs). * *p* < 0.05, ** *p* < 0.01, *** *p* < 0.001 compared with control. L-Rh3, 50 mg/kg; H-Rh3, 100 mg/kg; Gefitinib, 40 mg/kg.

To evaluate the effects of Rh3 on NSCLC tumor metastasis in vivo, we used A549 cells to build a lung metastasis model in nude mice. Compared with controls, tumor metastasis was significantly inhibited after treatment with Rh3 (Figure 10A). Furthermore, an immunofluorescence assay revealed that the fluorescence intensities of N-cadherin, p-ERK, ERK, and HIF-1α were significantly diminished, whereas that of E-cadherin was considerably enhanced after Rh3 treatment (Figure 10B,C). Western blotting showed that Rh3 enhanced E-cadherin expression and suppressed levels of N-cadherin, Vimentin, Snail, HIF-1α, p-ERK, ERK, and VEGF (Figure 11), which is in agreement with the experimental results in vitro. In addition, the results of the immunohistochemical assay corresponded strongly to the variations in the levels of proteins that were the results of the Western blotting (Figures S3 and S4). The findings suggest that Rh3 suppressed lung cancer metastasis in vivo.

Furthermore, Rh3 boosted p21 and p53 expression and decreased CyclinD1 and CDK4 expression (Figure 11). The in vivo observations of cell cycle-related protein expression were in agreement with the in vitro results.

### 2.7. Ginsenoside Rh3 induces Low Toxicity In Vivo

For the purpose of verifying the security of Rh3 for treating lung cancer in nude mice, the hematological parameters of white blood cells (WBCs), granulocytes (GRANs), and lymphocytes (LYM) were measured from the peripheral blood of all groups. There were no remarkable variations in the hematological parameters of WBCs, LYMs, or GRAN observed in the control and Rh3 treatment groups (Figure S5A–C). However, peripheral blood parameters were markedly lower as compared to the normal group in the gefitinib group. These findings showed no observable hematological toxicity for Rh3 but significant hematological toxicity for gefitinib (Figure S5D–G). However, there were no observed major weight changes between the Rh3-treated and control groups (Figure S5H). In addition, we observed normal histopathological appearance in the heart, liver, spleen, lungs, and kidneys of the Rh3 group after tissue slices were analyzed. Figure S5I shows the observed liver and spleen damage in the gefitinib group. To summarize, Rh3 exerted less toxicity in lung cancer treatment.

## 3. Discussion

Ginsenoside Rh3 is one of the rare ginsenosides that has been proven to possess powerful anti-tumor activity [38]. Nonetheless, the mechanism by which Rh3 inhibits the metastasis of lung cancer cells has not been explained. In the research presented here, we proved that Rh3 inhibited human lung cancer cell proliferation both in vitro and in vivo through a G1 phase block. In addition, through the ERK pathway, lung cancer cell metastasis was effectively suppressed both in vivo and in vitro. The cell viability assay revealed that Rh3 significantly suppressed lung cancer cell proliferation, with 160 µM Rh3 inhibiting 88.67 ± 2.5% of A549 cells and 83.33 ± 3.2% of PC9 cells. One of the methods proven to inhibit cancer cell proliferation is to block the cell cycle [39,40]. A range of protein kinases called CDKs regulate the cell cycle and these kinases are essential in tumor growth [41]. We demonstrated that Rh3 blocked A549 and PC9 cells in the G1 phase through enhancing p21 and p53 expression and decreasing levels of CyclinD1 and CDK4. In addition, in vivo experiments showed the same experimental results. High-dose Rh3 treatment induced cell cycle arrest and cytotoxicity, whereas low-dose Rh3 treatment exerted no significant effect on cell proliferation. The findings of the colony formation assay were in agreement with those of the MTT assay, and a low concentration of Rh3 exerted no significant effect on A549 and PC9 cell proliferation activity. Altogether, the above data suggested that the anti-cancer activity of low-dose Rh3 is exerted via modulation of the metastatic abilities of lung cancer cells and not via cytotoxicity.

The association of hypoxia and EMT has been identified in several cancers. Hypoxia promotes EMT in tumor cells by inducing EMT-related transcription factor expression [42,43]. Cancer cells undergoing EMT are not only more capable of acquiring invasiveness but also of remodeling the microenvironment to a state favorable for cancer metastasis [44]. In the present study, it was found that N-cadherin, Vimentin, and Snail expression was upregulated in both A549 and PC9 cells following CoCl_2_-induced hypoxia, whereas expression of E-cadherin was downregulated, indicating EMT procedure activation in lung cancer cells. Western blotting results showed that Rh3 significantly upregulated E-cadherin expression and suppressed N-cadherin, Vimentin, and Snail expression dose-dependently, suggesting that Rh3 decreased metastatic ability in lung cancer cells. The EMT procedure was inverted by Rh3 via the regulation of EMT-associated markers within hypoxic cells. Furthermore, our immunohistochemical and immunofluorescence results indicated that Rh3 exerted a regulatory effect on EMT-related markers, consistent with the results of our in vitro experiments.

We used network pharmacology to explore the potential targets of action of Rh3. Network pharmacology builds on the understanding of Rh3–target interactions, and network analysis can be used to observe the interrelationships between Rh3 and potential targets, allowing a clearer, more visual study of interactions between each node in a network. In addition, the molecular docking results revealed the docking sites for the binding of Rh3 to ERK target proteins and provided a theoretical basis for subsequent experiments.

In the last few years, metastasis suppression has attracted increasing interest as an active method for cancer prevention and targeted therapy. EMT has been associated with multiple complex signaling pathways [45]. Similarly, the regulation of HIF-1α during cellular responses has been implicated in angiogenesis and tumor development [46,47]. Hypoxia, CoCl_2_, and EGF induce the expression of HIF-1α [48]. We used CoCl_2_ to mimic hypoxia and enhance the stability of HIF-1α. Western blotting results revealed that HIF-1α, p-ERK/ERK, and VEGF expression were significantly elevated after the induction of hypoxia, indicating that hypoxia induced the accumulation of p-ERK and HIF-1α and regulated the level of VEGF in both A549 and PC9 cells. Following Rh3 treatment, HIF-1α, p-ERK, ERK, and VEGF levels were regulated and reduced via a dose-dependent approach. Similar to these findings, our research confirmed that the combined induction of U0126 or ERK knockdown with Rh3 significantly reduced the metastasis of lung cancer cells, which indicated that Rh3 suppressed lung cancer cell metastasis by reducing p-ERK expression.

We found that 50 mg/kg and 100 mg/kg Rh3 significantly inhibited lung cancer metastasis in vivo. The levels of G1 phase-associated proteins, metastasis-associated proteins, p-ERK/ERK, HIF-1α, and VEGF that were regulated after Rh3 treatment were observed by means of Western blotting and immunohistochemical analyses, and the results were consistent with our in vitro observations. In addition, hematoxylin and eosin (H&E) staining confirmed that Rh3-treated mice experienced no vital organ-associated toxicity. To recap, we demonstrated that Rh3 blocks the G1 phase and correlates with the EMT-sensitive ERK signaling pathway. In a lung cancer metastasis model, Rh3 significantly inhibited metastasis with few side effects. We believe that our study will provide novel ideas regarding the use of Rh3 as a promising clinical anti-tumor agent and also highlight ERK as a potential molecular target for Rh3 and cancer metastasis.

## 4. Materials and Methods

### 4.1. Materials

Ginsenoside Rh3 ([HPLC] ≥ 98%) was obtained from the Biomedical Research Institute of Northwest University (Xi’an, Shaanxi, China—lot number: 20,210,723; validity period extending until July 2023). RPMI 1640 was purchased from HyClone (Logan, UT, USA). Methylthiazolyldiphenyl tetrazolium bromide (MTT) and cobalt chloride (CoCl_2_) were purchased from Beyotime Biotechnology (Shanghai, China). Trypsin, penicillin–streptomycin (pen–strep), and crystal violet were purchased from Solarbio Science & Technology Co., Ltd. (Beijing, China). Fetal bovine serum (FBS) was purchased from Biological Industries (Cromwell, CT, USA). U0126 was purchased from MedChemExpress. Bouin’s solution was purchased from Phygene (Fuzhou, Fujian, China). The list of antibodies used is shown in Supplementary Table S1.

### 4.2. In Vitro Cytotoxicity Assay

Cell viability was measured using the MTT method. In brief, tumor cells (1 × 104 cells/well) were seeded in 96-well plates and cultured with or without drugs for 24 h and 48 h. Next, 50 μL of 0.5 mg/mL MTT labeling reagent was added to each well, and the cells were incubated for 2 h in a cell incubator. Afterward, 150 μL of DMSO was added to each well and absorbance was measured at 490 nm using a microplate reader (Bio-Tek Instruments, Inc., Winooski, VT, USA). We calculated the average of five repetitions for each concentration.

### 4.3. Cell Migration and Invasion Assays

Cell wound assays were used to examine cell migration. The cells were incubated in 6-well plates in a fused monolayer. Afterward, using a 200 μL pipette tip to scrape off the monolayer, the plates were washed three times with PBS to remove the floating cells. To induce hypoxic conditions, 100 μM CoCl_2_ was used, and the cells were incubated for 24 h in the absence or presence of Rh3 (20 μM and 40 μM). ImageJ software (National Institutes of Health) was used to calculate the percentages of cell migration in the acquired images.

For the Transwell migration assay, hypoxic conditions were induced in cells using 100 μM CoCl_2_, and the cells were subsequently inoculated into the upper compartment, with 500 μL of medium with 10% FBS added to the lower chamber. Migrating cells were fixed with 4% paraformaldehyde on the lower surface of the filter membrane and treated at room temperature with 0.1% crystal violet. Cells were viewed using an inverted microscope and five fields of vision were randomly selected for each hole for the acquisition of images.

To study cell invasion, the same experiment was performed as for the cell migration assay, except that 50 μL of matrix gel was pre-coated in the upper compartment.

### 4.4. Flow Cytometry Analysis

Cells were distributed in a 6-well plate and incubated for 24 h. Afterward, the cells were reprocessed with ginsenoside Rh3 for another 24 h. Subsequently, the cells were collected by centrifugation and fixed with ice-cold 70% ethanol overnight. The fixed cells were treated with phosphate-buffered saline (PBS) containing 0.5 mg/mL ribonuclease A and stained with 50 mg/mL propidium iodide (PI) in the dark for 30 min. The fluorescence intensity of a single nucleus was measured by flow cytometry, and the data were analyzed using FlowJo software.

### 4.5. Western Blotting

Monolayers of A549 cells and PC9 cells, following various treatments, were lysed in the RIPA buffer containing proteases and phosphatase inhibitors (Solarbio). Total protein was extracted, and the total protein content was quantified using a BCA protein assay kit. Equal amounts of sample proteins were loaded onto sodium dodecyl sulfate–polyacrylamide gel electrophoresis (SDS–PAGE). After electrophoresis, samples were transferred to polyvinylidene fluoride (PVDF) membranes. Next, the membranes were blocked with skimmed milk powder in Tris-buffered saline and Tween 20 (TBST) buffer for 2 h and left overnight at 4 °C to bind with primary antibodies. After being washed five times with TBST buffer, a diluted secondary antibody was added, and the membranes were incubated for 1 h at room temperature. The bands were visualized using enhanced chemiluminescence (ECL) reagent.

### 4.6. RNA Isolation and RT-qPCR

After treatment of the posterior lung adenocarcinoma cells with CoCl_2_ and ginsenoside Rh3, the cells were lysed using TRIzol reagent and total RNA was isolated. Next, complementary DNA (cDNA) was synthesized using a RevertAid First Strand cDNA Synthesis Kit (Thermo Fisher Scientific, Waltham, MA, USA), and a polymerase chain reaction (PCR) was performed using a quantitative reverse transcriptase PCR (qRT-PCR) system (Bio-Rad, Hercules, CA, USA). The RNA expression of the β-actin gene was used to normalize the relative RNA expression of each gene. The list of primers used is shown in Table 1.

### 4.7. siRNA Transfection Analysis

The ERK siRNA (sense: 5′-GCUGCAUUCUGGCAGAAAUTT-3′; antisense: 5′-AUUUCUGCCAGAAUGCAGCTT-3′) was chemically synthesized by GenePharma (Shanghai, China). Following the manufacturer’s instructions, cells with 10 pM siRNA duplexes were transfected in 6-well plates to be used with Lipofectamine™ 2000 (Invitrogen, Carlsbad, CA, USA).

### 4.8. Analysis of Metastasis in Nude Mice

The experiments on animals were conducted in accordance with the guidelines of the Institutional Animal Care and Use Committee (NWU-AWC-20210304M). Male BALB/c nude mice (28–35 days old, 14–18 g) were obtained from GemPharmatech Co., Ltd. (Nanjing, China).

For the pulmonary metastasis model, mice were inoculated with 1 × 106 cells in the tail vein. The mice were randomly divided into four groups (*n* = 8) and treated with gefitinib (40 mg/kg) and Rh3 (50 mg/kg, 100 mg/kg). The mice were euthanized four weeks later and the lungs were stained using Bouin’s solution and observed for lung metastatic nodules [49].

### 4.9. Network Construction and Molecular Docking

The structure of Rh3 was obtained in PubChem, (https://pubchem.ncbi.nlm.nih.gov/ accessed on 25 May 2021). The target genes related to the selected components were identified using the PharmMapper server database, (http://www.lilab-ecust.cn/pharmmapper/ accessed on 25 May 2021) with the “Homo sapiens” species setting. Gene information, including gene ID and organism information, was retrieved from the UniProt database, (https://www.uniprot.org/ accessed on 29 May 2021). To further analyze the molecular mechanism of Rh3, we used online tools, (STRING; https://www.string-db.org/ accessed on 3 June 2021) to predict the interactions between target genes, with confidence >0.96 as the cut-off criterion, and hide disconnected nodes in the network. Next, we built compounds and target gene networks using Cytoscape software, (version 3.8.2, Seattle, DC, USA) [50]. Docking simulations were performed using Maestro software to validate the results of the network pharmacology-based analysis and obtain docking scores which provided a theoretical basis for subsequent experiments [51].

### 4.10. Statistical Analysis

Three repetitions of all the experiments were performed. The Statistical Package for the Social Sciences (SPSS; version 23.0, IBM Inc., Armonk, NY, USA) software was used for statistical analysis. One-way analysis of variance (ANOVA) with multiple groups was used to determine statistical significance, and a *p*-value < 0.05 was considered significant. Results are presented as means ± standard deviations (SDs) obtained from three independent samples or more.

## Figures and Tables

**Table 1:** Sequences of primers used in qRT-PCR.

Genes	Forward Primer (5′–3′)	Reverse Primer (5′–3′)
β-catenin	GGGATTGGCTTTAGGCCTGT	GAAATTGCCGTAGCGGGTTC
E-cadherin	GACGCCATCAACACCGAGTT	AAATTGCCAGGCTCAATGAC
N-cadherin	GGTGGAGGAGAAGAAGACCAG	GGCATCAGGCTCCACAGT
Vimentin	GACGCCATCAACACCGAGTT	CTTTGTCGTTGGTTAGCTGGT
Snail	CTTCCAGCAGCCCTACGAC	CGGTGGGGTTGAGGATCT
HIF-1α	TTATCAACACCCCCTGCGACCT	GCCACATACTCCGTCAGAAAGC
VEGF	CTCATCAGTTGCCACTTCCACATA	AGCAATTCATCTGTGCTTTCATGTC
ERK	TCACAGGTACAGGGATGAGGACAC	CAAAGCACAGCAATGTCCTGAAG

## Data Availability

All data are contained within the article and the Supplementary Materials.

## Supplementary Materials

The following supporting information can be downloaded at: https://www.mdpi.com/article/10.3390/ph15060758/s1, Figure S1: Cytotoxicity of CoCl_2_ treatment of A549 and PC9 cells after 24 h as determined by MTT assay; Figure S2: Target prediction of ginsenoside Rh3 based on the PharmMapper database and bioinformatics analysis; Figure S3: IHC staining of EMT-related proteins (N-cadherin, E-cadherin, Vimentin, Snail) in the lungs of mice after receiving A549 cells in the tail vein; Figure S4: IHC staining of HIF-1α, ERK, p-ERK, and VEGF proteins in the lungs of mice after receiving A549 cells in the tail vein; Figure S5: Rh3 induces low toxicity in vivo; Table S1: Information on antibodies.
