# Supplementary material for: Ginsenoside Rh3 Inhibits Lung Cancer Metastasis by Targeting Extracellular Signal-Regulated Kinase: A Network Pharmacology Study
Source: Pharmaceuticals (Basel). 2022 Jun 17;15(6):758. doi: 10.3390/ph15060758 (PMC9229598; doi:10.3390/ph15060758)
Supplement: Supplementary file 1 [file pharmaceuticals-15-00758-s001.zip › pharmaceuticals-1739439-SI.pdf]

Ginsenoside Rh3 Inhibits Lung Cancer Metastasis by Targeting Extracellular Signal-Regulated Kinase: a Network Pharmacology Study

Xiaodan Xue [1,2,3,†], Yannan Liu [1,2,3,†], Linlin Qu [1,2,3,†], Cuiying Fan [4], Xiaoxuan Ma [1,2,3], Pingkai OuYang [5], Daidi Fan [1,2,3,*]

1 Shaanxi Key Laboratory of Degradable Biomedical Materials, School of Chemical Engineering, North-west University, Taibai North Road 229, Xi'an, Shaanxi 710069, China

2 Shaanxi R&D Center of Biomaterials and Fermentation Engineering, School of Chemical Engineering, Northwest University, Taibai North Road 229, Xi'an, Shaanxi 710069, China

3 Biotech. & Biomed. Research Institute, Northwest University, Taibai North Road 229, Xi'an, Shaanxi 710069, China

4 Xi'an Giant Biotechnology Co., Ltd, Xi'an, Shaanxi, China College of biotechnology and Pharmaceutical Engineering, Nanjing Tech University, Nanjing 211816, China

5 College of Biotechnology and Pharmaceutical Engineering, Nanjing Tech University, Nanjing 211816, China

* Correspondence: fandaidi@nwu.edu.cn; Tel.: +86-29-88305118

† These authors contributed equally to this work.

1. *Supplementary Methods*

1.1. Cell lines and cell culture

Cancers cell lines A549 and PC9 were purchased from the Shanghai Institute of Cell Biology. All cell lines were free of microbial contamination, including mycoplasma. A459 cells were grown in the RPMI 1640 medium supplemented with 10% FBS and pen/strep antibiotics at 37°C in the presence of 5% CO_2.

1.2. Cell colony-forming assay

Cells were seeded in a 6-well plate at a density of 500 cells/well. After adhesion, the cells were treated with ginsenoside Rh3 for 24 h, following which the culture medium was changed every 2 days with a drug-free medium. The formed cell colonies were fixed with methanol and subsequently stained with crystal violet staining solution. Finally, the images were acquired and the number of colonies was counted.

1.3. Immunofluorescence staining

Tissue samples were fixed with 4% paraformaldehyde on glass coverslips to fully fix and dehydrate before sectioning, closed with 5% bovine serum albumin for 1 hour at room temperature, and then incubated with the corresponding primary antibodies overnight at 4 °C. After washing with PBS buffer, the sections were further incubated with fluorescent secondary antibodies for 2 hours at room temperature and then stained at room temperature. Cover slips were mounted on slides containing anti-fluorescence quencher and observed under an Olympus confocal microscope (Tokyo, Japan). Three

of the final microscope images were randomly selected for quantitative analysis.

1.4. Hemogram assay and measurement of biochemical parameters

Blood was collected from each group on day 28. Peripheral blood was taken from the retro-orbital plexus of mice and transferred to tubes containing ethylenediaminetetraacetic acid. The white blood cell (WBC) count, lymphocyte (LYM) count and granulocyte (GRAN) count of each specimen were measured using a fully automated hematology analyzer (HC2200, Meiyilin, China).

Blood samples were centrifuged at 1000 g for 15 min at 4 °C to obtain serum. Important indicators of liver function (ALT) and important indicators of renal function (Urine Creatinine, Uric acid and BUN) were measured by ELISA kits according to the instructions of the manufacturer (Shanghai Enzyme Biotechnology Co., Ltd., Shanghai, China).

1.5. Histopathology and Immunohistochemistry assay

Tumor tissue and major organs (heart, liver, spleen, lung and kidney) were fixed in 10% formalin and embedded in paraffin wax. The paraffin was then cut into 5 μm slices. The nuclei were stained with 10% hematoxylin and the cytoplasm was stained with 1% eosin. For immunohistochemistry, the tissue samples were fixed, paraffin-embedded, sectioned at 5 μm thickness, and subsequently stained according to the standard immunohistochemistry protocol. The antibodies and their concentrations used are shown in Supplementary Table S1.

2. Supplementary Figures and Tables

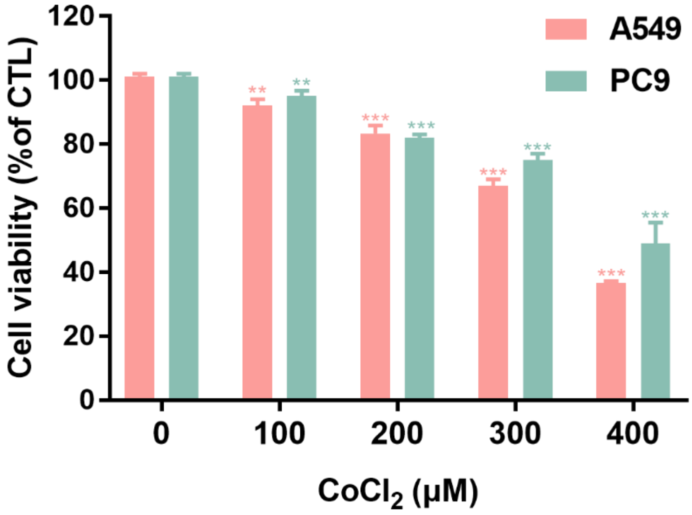

Figure S1. Cytotoxicity of $CoCl_2$ (0–400 μM) treatment on A549 and PC9 cells after 24 h were determined by MTT assay. $^{**}P < 0.05$; $^{***}P < 0.01$ compared with control.

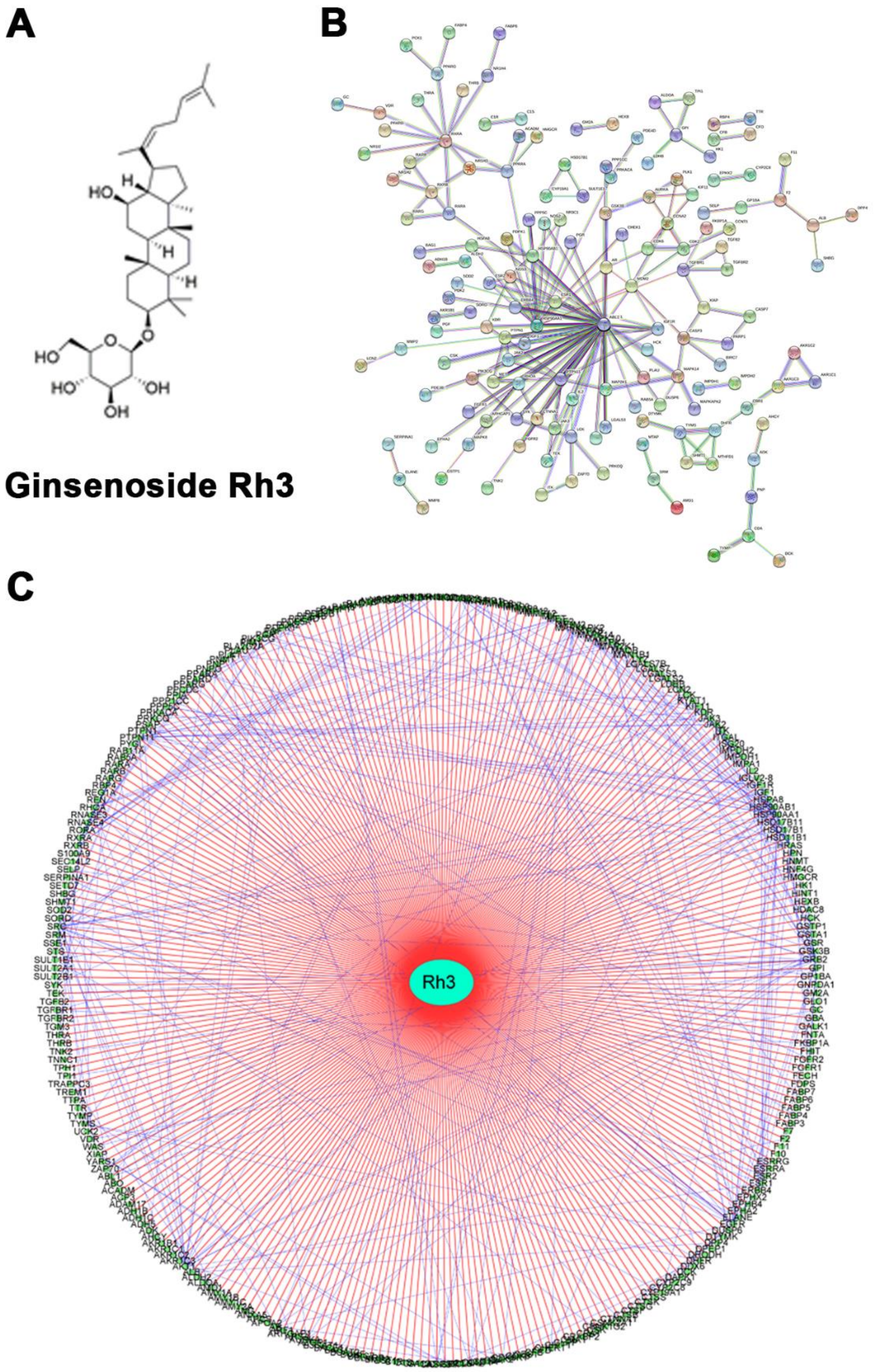

Figure S2. Target prediction of ginsenoside Rh3 based on Pharmmapper database and bioinformatics analysis. (A) Molecular structural formula of ginsenoside Rh3; (B) PPI network diagram of target proteins; (C) Network pharmacology.

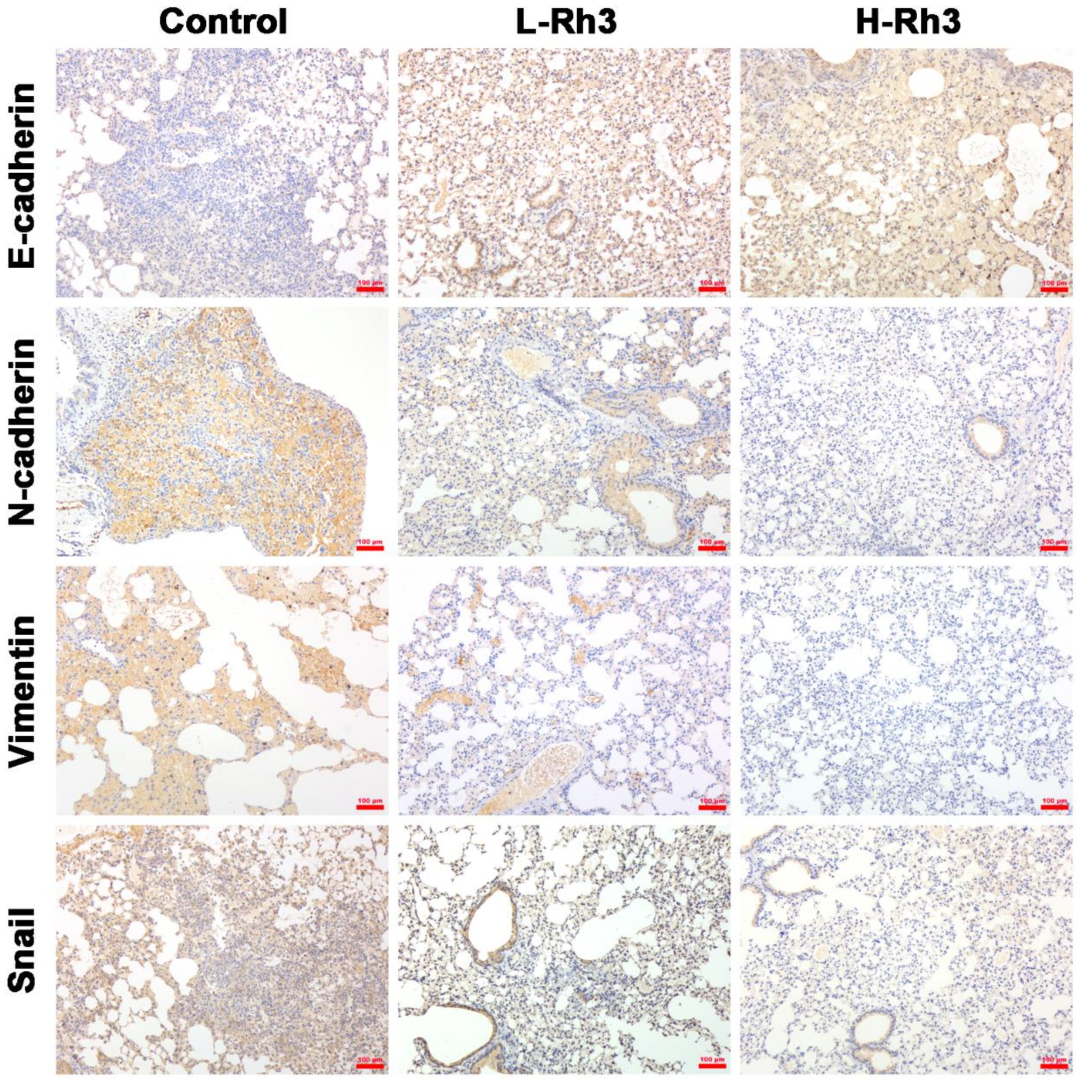

Figure S3. IHC stating of EMT related protein (N-cadherin, E-cadherin, Vimentin, Snail) in lung of mice after receiving A549 cells in the tail vein. L-Rh3, 50 mg/kg, H-Rh3, 100 mg/kg, Gefitinib, 40 mg/kg.

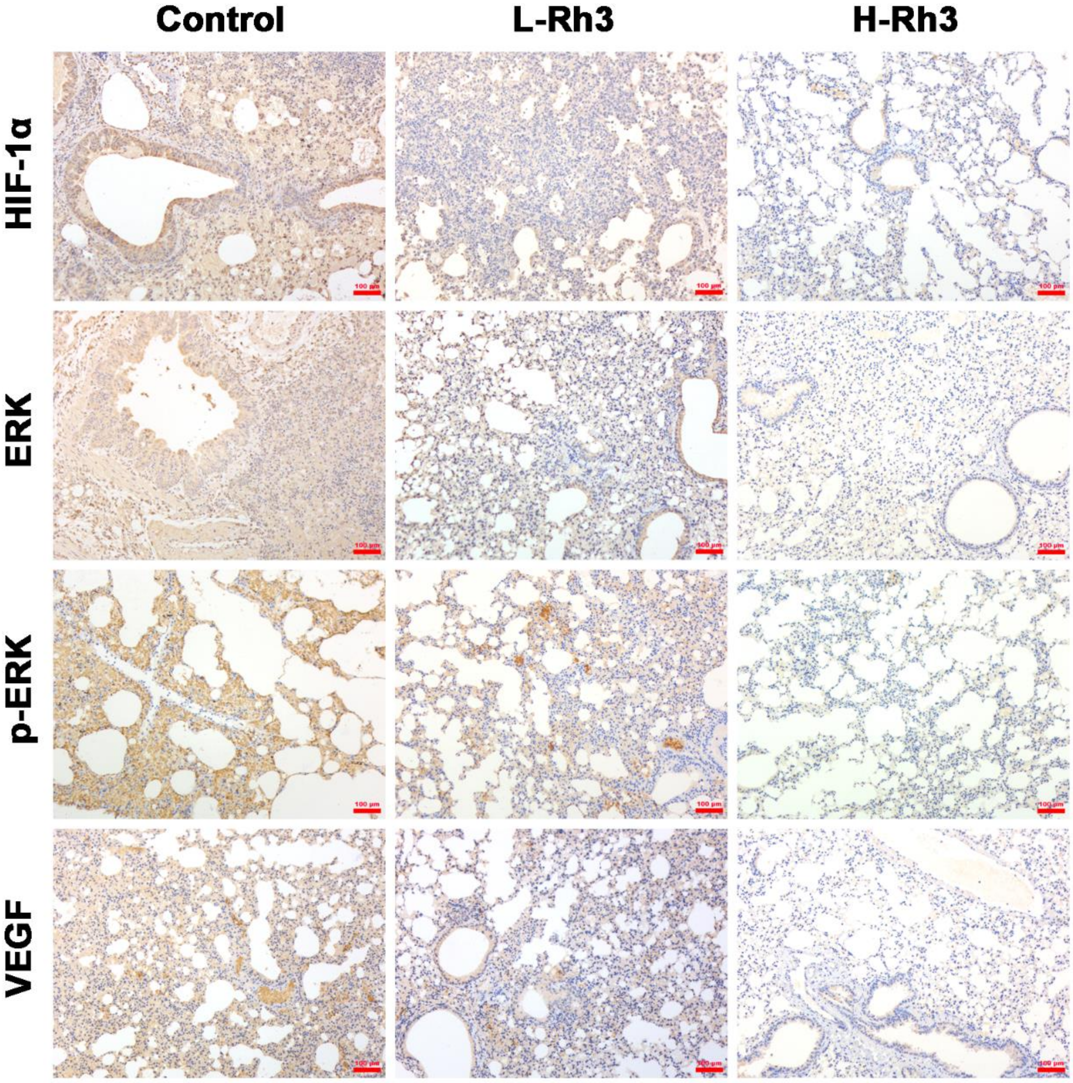

Figure S4. IHC stating of HIF-1α, ERK, p-ERK, VEGF protein in lung of mice after receiving A549 cells in the tail vein. L-Rh3, 50 mg/kg, H-Rh3, 100 mg/kg, Gefitinib, 40 mg/kg.

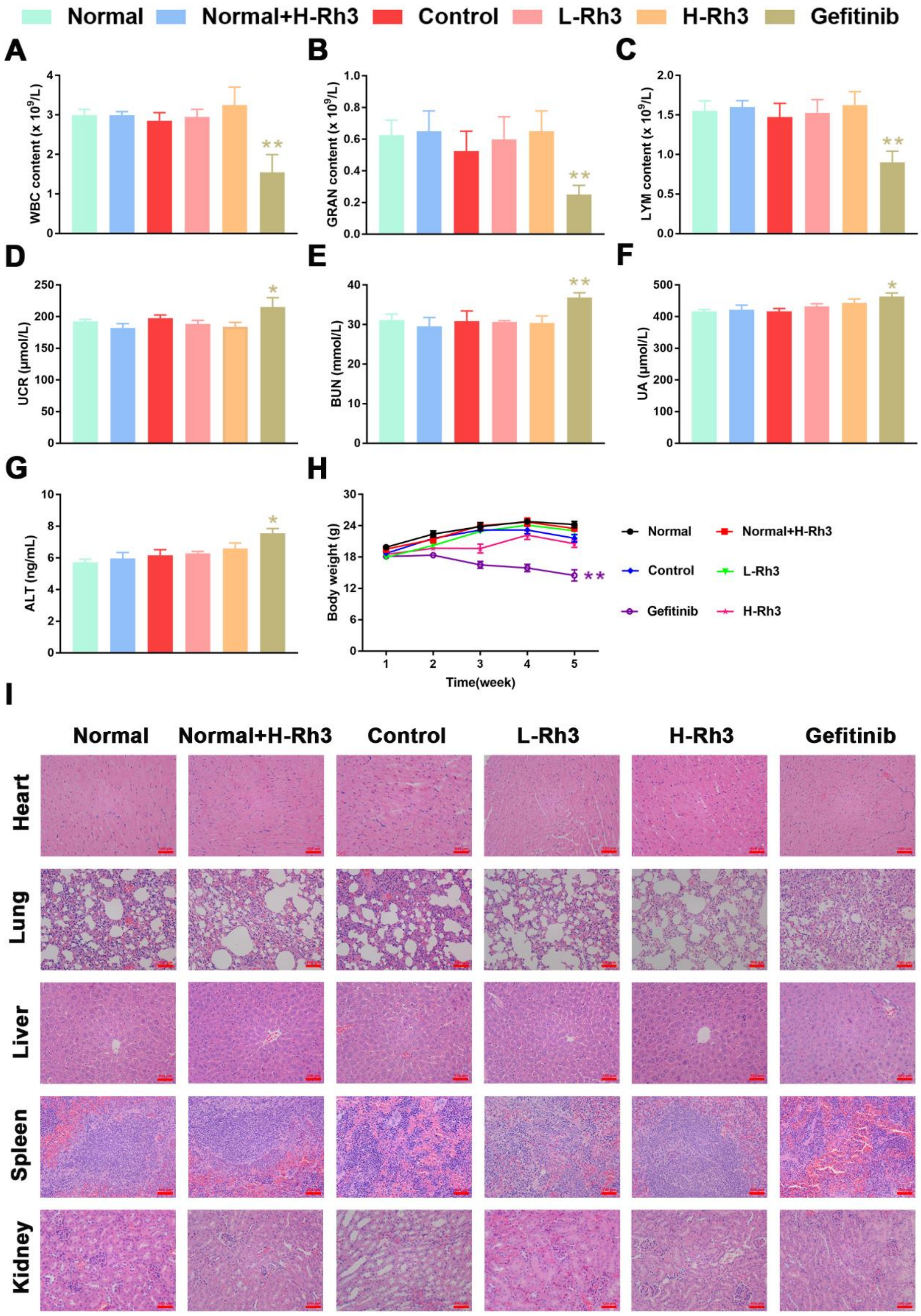

Figure S5. Rh3 induces low toxicity *in vivo*. (A) WBC content of blood in mice after receiving A549 cells in the tail vein. (B) GRAN content of blood in mice after receiving A549 cells in the tail vein. (C) LYM content of blood in mice after receiving A549 cells

in the tail vein. (D) UCR content of blood in mice after receiving A549 cells in the tail vein. (E) BUN content of blood in mice after receiving A549 cells in the tail vein. (F) UA content of blood in mice after receiving A549 cells in the tail vein. (G) ALT content of blood in mice after receiving A549 cells in the tail vein. (H)Body weight variation of mice after receiving A549 cells in the tail vein. (I) H&E staining of main organ (heart, liver, spleen, lung, and kidney) in mice after receiving A549 cells in the tail vein. Data are shown as mean ± SD, $^{*}P < 0.05$, $^{**}P < 0.01$ compared with the normal group. L-Rh3, 50 mg/kg, H-Rh3, 100 mg/kg, Gefitinib, 40 mg/kg.

Table S1 Information of antibodies

Antibody	Immunofluorescence	Immunohistochemistry	Western blot	Manufacturer	Molecular Weight (kDa)	Cat. Number
HIF-1α	1:500	1:100	1:1000	Abcam	83	Ab51608
p-ERK	1:200	1:800	1:1000	CST	42	#8544
ERK	1:100	1:500	1:4000	PTG	44	16443-1-AP
CyclinD1	---	---	1:2000	PTG	34	26939-1-AP-
CDK4	---	---	1:4000	PTG	34	11026-1-AP
P21	---	---	1:3000	PTG	21	10355-1-AP
P53	---	---	1:6000	PTG	53	100442-1-AP
N-Cadherin	1:300	1:4000	1:10000	PTG	130	22018-1-AP
E-Cadherin	1:300	1:4000	1:10000	PTG	120	20874-1-AP
Vimentin	---	1:10000	1:10000	PTG	54	10366-1-AP
Snail	---	1:800	1:1000	PTG	29	13099-1-AP
VEGF	---	1:500	1:2000	PTG	25	19003-1-AP
β-actin	---	---	1:40000	PTG	42	20536-1-AP
